# Exploration of the Temperature Sensing Ability of La_2_MgTiO_6_:Er^3+^ Double Perovskites Using Thermally Coupled and Uncoupled Energy Levels

**DOI:** 10.3390/ma14195557

**Published:** 2021-09-24

**Authors:** Thi Hong Quan Vu, Bartosz Bondzior, Dagmara Stefańska, Przemysław J. Dereń

**Affiliations:** Institute of Low Temperature and Structure Research, Polish Academy of Sciences, Okólna 2, 50-422 Wrocław, Poland; q.vu@intibs.pl (T.H.Q.V.); b.bondzior@intibs.pl (B.B.); d.stefanska@intibs.pl (D.S.)

**Keywords:** double perovskites, La_2_MgTiO_6_, erbium, luminescence, luminescent thermometry

## Abstract

This work aimed to explore the temperature-sensing performance of La_2_MgTiO_6_:Er^3+^ double perovskites based on thermally coupled and uncoupled energy levels. Furthermore, the crystal structure, chemical composition, and morphology of the samples were investigated by powder X-ray diffraction, energy-dispersive X-ray spectroscopy, and scanning electron microscopy, respectively. The most intense luminescence was observed for the sample doped with 5% Er^3+^. The temperature-dependent emission spectra of La_2_MgTiO_6_:5% Er^3+^ were investigated in the wide range of 77–398 K. The highest sensitivity of the sample was equal to 2.98%/K corresponding to the thermally coupled energy level ^2^H_11/2_ → ^4^I_15/2_ and ^4^S_3/2_ → ^4^I_15/2_ as compared to 1.9%/K, obtained for the uncoupled energy level ^2^H_11/2_ → ^4^I_15/2_ and ^2^H_9/2_ → ^4^I_15/2_. Furthermore, the 300 K luminescent decay profiles were analyzed using the Inokuti–Hirayama model. The energy transfer among Er^3+^ ions was mainly regulated by the dipole–dipole mechanism. The critical transfer distance R_0_, critical concentration C_0_, energy transfer parameter C_da_, and energy transfer probability W_da_ were 9.81 Å, $2.53\times 10^{20}$ ions·cm^−3^, $5.38\times 10^{-39}\;$cm^6^·s^−1^, and 6020 s^−1^, respectively.

## 1. Introduction 

Double perovskite compounds (DP) are among the most intensely studied materials because of their interesting chemical and physical properties, as well as their diverse applications stemming from the compositional flexibility of their structure. La_2_MgTiO_6_ (LMT), a representative of the double perovskite family, has recently been extensively investigated as a host of many lanthanide ions [1,2,3]. The energy transfer process from TiO_6_ groups to lanthanides ions takes place very efficiently [3]. Erbium ions have been widely applied in eye-safe lasers, optical communications, or lighting owing to their broad spectral range spans in the ultraviolet, visible, and near-infrared regions [4,5,6]. The most universal approach for LMT is a white-light conversion material related to emission from Sm^3+^ and Eu^3+^ [7], Mn^4+^ and Yb^3+^ [8], Bi^3+^ [9], Eu^3+^ [10], Eu^2+^ [11], Mn^4+^ [12,13,14,15], and Gd^3+^, Bi^3+^, Sm^3+^, and Eu^3+^ ions [16]. Furthermore, an ample potential for optical thermal sensing has recently been demonstrated through these studies including LMT:Pr^3+^ [17], LMT:Eu^3+^ [3], and LMT:Nd^3+^ [2].

Temperature is one of the most important physical parameters which is present in many fields of science and life. In the last few decades, there has been an explosion of research on such optical thermometry [17,18,19,20,21,22,23] to fulfil the requirements of fast growth in a myriad of fields including chemistry, physics, biomedicine, and industry. Indeed, noncontact thermometers possess numerous advantages, such as remote, handy, and precise application, high spatial resolution, and a broad operating temperature range as compared to the conventional thermometers [24]. What is more, they can operate in harsh conditions, e.g., high pressure, corrosive, and very low or high temperature, where the traditional techniques are infeasible [22]. To design luminescent thermometry, taking into account the reliance of peak position, emission intensity, and lifetime when elevating temperature, the sensitivity of thermometers should be estimated. The ratio of emission intensity between two distinct transitions is preferable since it is an external interference-independent factor [24]. It is worth noting that there has only been one investigation into LMT:Er^3+^ published recently with regard to temperature readout application. However, it was synthesized using the conventional solid-state method, the working temperature range was quite narrow, and it was only focused on thermally coupled levels [25].

Therefore, a comprehensive investigation of the temperature-sensing ability of LMT:Er^3+^ synthesized via the coprecipitation method was conducted by exploring both thermally coupled and uncoupled energy levels. The well-known thermally coupled levels correspond to the transitions ^2^H_11/2_ → ^4^I_15/2_ and ^4^S_3/2_ → ^4^I_15/2_, which are separated from each other by 592 cm^−1^. The energy gap between these levels should be neither too close to overlap nor too far to be easily populated by the other; moreover, it should be in the range from 200 cm^−1^ to 2000 cm^−1^ [26]. Nonetheless, uncoupled levels, which are related to the phonon-assisted process, have also gained significant attention for temperature sensing [18,27,28]. This is due to the fact that the phonon-assisted process enhanced by elevating temperature is also related to absorption, emission, and energy transfer. Thus, the exploration of all possibilities including thermally coupled and uncoupled levels is crucial to choose the highest sensitivity with the lowest uncertainty.

The aim of this work was to explore the temperature sensing performance of a novel double perovskite LMT:Er^3+^ as a function of the ratio of emission intensity of thermally coupled and uncoupled energy levels of Er^3+^ ions. Additionally, the structural, morphological, and spectroscopic properties including absorption and emission spectra were investigated. The energy transfer mechanism among Er^3+^ ions and the characteristics of luminescent decay kinetics were clarified using the Inokuti–Hirayama model. 

## 2. Experimental 

### 2.1. Synthesis

La_2_MgTiO_6_:x Er^3+^ (where x = 0%, 0.1%, 0.5%, 1%, 3%, 5%, or 7%) was successfully obtained via the coprecipitation method. The procedure was calculated for 0.5 g of sample. The erbium ions were incorporated into the lanthanum site in the structure. All precursors used for the syntheses were purchased from the Alfa Aesar company (Kandel, Germany), including lanthanum acetate (La(CH_3_COO)_3_∙1.5H_2_O, 99.9%), magnesium nitrate (Mg(NO_3_)_2_∙6H_2_O, 99.97%), titanium isopropoxide (Ti(C_3_H_7_O)_4_, 95%), erbium oxide (Er_2_O_3_, 99.99%), nitric acid (HNO_3_, 65%, POCH), and ammonium hydroxide (NH_4_OH, 25%, CHEMPUR). The synthesis method, annealing time, and sintering temperature were described in previous publications [2,3]. Firstly, the proper amounts of erbium oxide and titanium isopropoxide were separately diluted in nitric acid solution. Afterward, the prepared solution of titanium isopropoxide was added into the mixture of the aqueous solution containing lanthanum, magnesium, and erbium ions (for doped samples). However, it should be emphasized that a 10% excess amount of magnesium ions was applied due to the likelihood of the sublimation of magnesium ions during the high-temperature sintering process. To obtain the precipitate, a solution of ammonium hydroxide (1 mL) was added. The obtained precipitate was dried at 80 °C for 24 h on a heater. Then, it was sintered at 600 °C for 12 h in a porcelain crucible. The second annealing was conducted at 1300 °C for 8 h in a corundum crucible in air. Grinding for a few minutes was necessary after each step of annealing.

### 2.2. Characterization 

The powder X-ray diffractions of all samples were measured on an X’Pert ProPANalytical (PANalytical, Almelo, The Netherlands) using an X-ray diffractometer with Cu K_α_ radiation (λ = 1.54056 Å) in a 2θ range from 10° to 90° with a step size of Δ2θ = 0.02°. The morphology and chemical composition of LMT:5% Er^3+^ were investigated using a scanning electron microscope FEI NOVA NanoSEM230 (FEI, Hillsboro, OR, USA). To determine the absorption spectrum of LMT:7% Er^3+^, a Varian Cary 5E UV/Vis–NIR spectrophotometer (Varian Incorporation, Palo Alto, CA, USA) was used. The 300 K emission spectra of all samples were obtained using a Hamamatsu Photonic multichannel analyzer PMA-12 (Hamamatsu Photonics K.K, Shizuoka, Japan) along with a BT-CCD linear image sensor. Furthermore, a McPherson spectrometer linear PIXcel detector (McPherson Incorporation, Chelmsford, MA, USA) equipped with an 808 diode laser was used for infrared emission measurement. The 300 K emission decay profiles were recorded with a Lecroy digital oscilloscope (Teledyne Technologies, New York, NY, USA) using an excitation source of Nd:YAG. The thermal quenching measurements were measured using the Hamamatsu Photonic multichannel analyzer PMA-12 equipped with the BT-CCD linear image sensor. The temperature of the samples was controlled by a Linkam THMS 600 Heating/Freezing Stage (The McCRONE Group, Westmont, IL, USA).

## 3. Results and Discussion

### 3.1. Structural and Morphological Characterization

**Figure 1:**
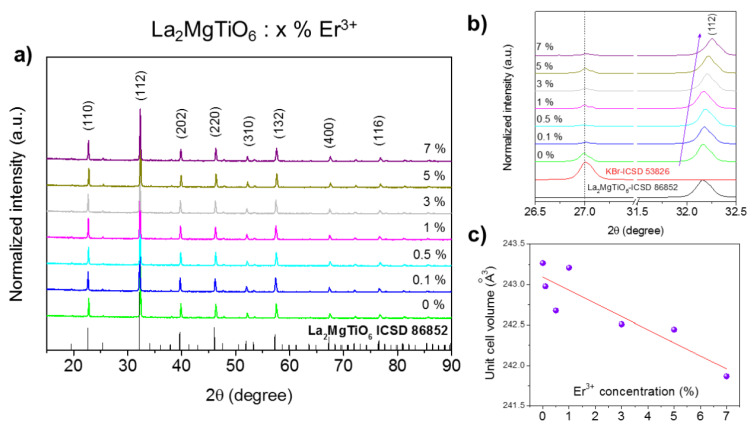
(**a**) X-ray powder diffraction of La_2_MgTiO_6_:x Er^3+^, (x = 0%, 0.1%, 0.5%, 1%, 3%, 5%, or 7%); (**b**) position correction of main diffraction peak of La_2_MgTiO_6_ with the help of KBr; (**c**) changing of unit cell volume as a function of Er^3+^ concentration (in the inset).

The XRD patterns of all prepared samples and the standard pattern of La_2_MgTiO_6_ (ICSD no. 86852) are shown in Figure 1. All samples crystallized in an orthorhombic structure with space group *Pbnm* (62) with the following lattice parameters: a = 5.5609 (1), b = 5.5738 (1), c = 7.8623 (2), V = 243.69 Å^3^, and Z = 4 [2,3]. No impurity traces were found. The main diffraction lines of the samples observed at 2θ values of 22.72°, 32.33°, 39.87°, 46.30°, 52.16°, 57.59°, 67.52°, and 76.82° were assigned to the lattice planes (hkl) of (110), (112), (202), (220), (310), (132), (400), and (116), respectively [29].

Due to the similarity of charge and ionic radius (121.6 pm for La^3+^ and 106.2 pm for Er^3+^ with the same coordination number, CN = 9 [30]), it can be assumed that Er^3+^ ions most likely substituted for La^3+^ ions located in the *C_s_* symmetry site. Therefore, the replacement of smaller Er^3+^ ions with larger La^3+^ ions resulted in the contraction of the crystal structure at higher dopant concentration. In order to prove our hypothesis, the XRD of all samples was measured again with a pure powder of potassium bromide (KBr, ICSD no. 53826), and the position of diffraction lines (2θ = 32.3°) of La_2_MgTiO_6_ was corrected (Figure 1b). The observed right-shifting of the diffraction peaks (Figure 1b) and the shrinkage of the unit cell volume with an increase in Er^3+^ concentration (Figure 1c) confirmed the above assumption. The concentration-dependent lattice parameters are shown in Figure S1 (Supplementary Materials).

**Figure 2:**
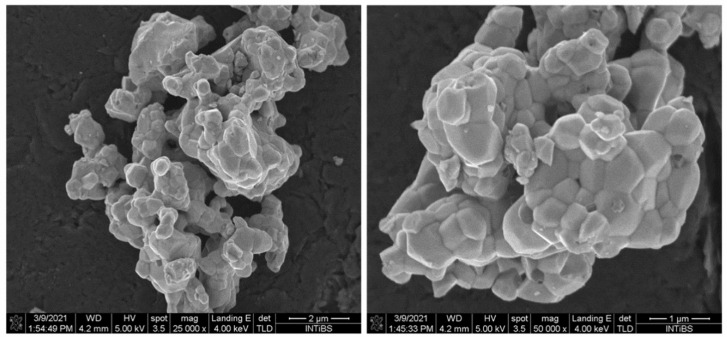
SEM images of sample La_2_MgTiO_6_:5% Er^3+^.

**Figure 3:**
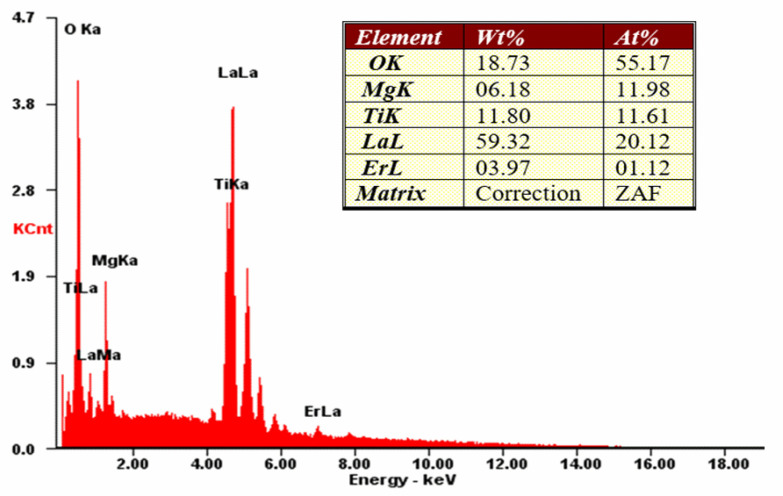
Results of EDS X-ray microanalysis of La_2_MgTiO_6_:5% Er^3+^.

The morphology and the energy-dispersive spectroscopy (EDS) X-ray microanalysis of LMT:5% Er^3+^ are presented in Figure 2 and Figure 3, respectively. From the SEM images, no individual grains were observed, and the grain size ranged from 0.5 to 1.0 mm, much smaller than that (5 μm) obtained via the conventional solid-state method [25]. It is clearly shown that small grains were strongly aggerated to form bigger objects. From the EDS measurements, all elemental compositions of LMT:5% Er^3+^ were identified and quantified including La (59.32 wt.%), Er (3.97 wt.%), Mg (6.18 wt.%), Ti (11.8 wt.%), and O (18.73 wt.%). These values are consistent with the general chemical formula: La (58.8 wt.%), Er (3.73 wt.%), Mg (5.41 wt.%), Ti (10.67 wt.%), and O (21.39 wt.%).

### 3.2. Absorption Spectrum

**Figure 4:**
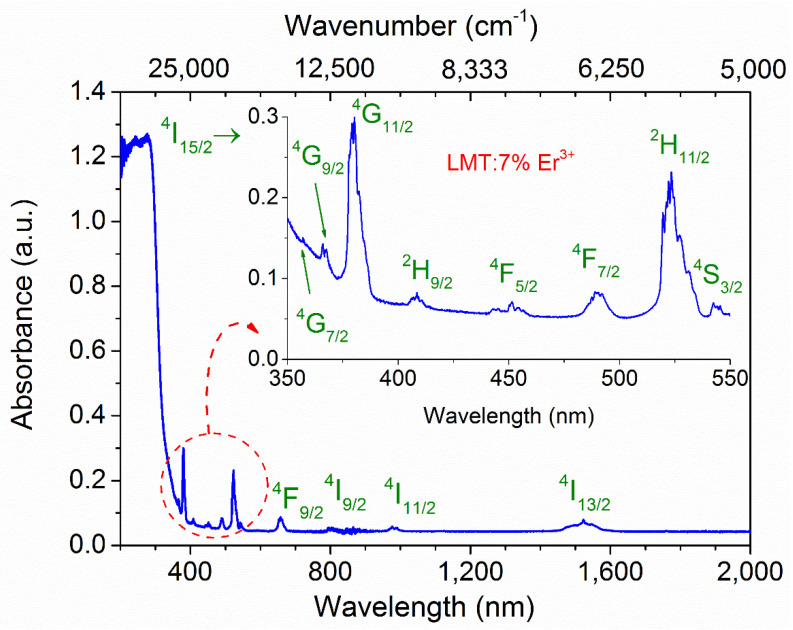
Absorption spectrum of La_2_MgTiO_6_:7% Er^3+^ at room temperature.

The absorption spectrum of LMT:7% Er^3+^ in the range of 200–2000 nm, shown in Figure 4, exhibited typical transitions for Er^3+^ ions. Intense absorption bands starting from 200 nm up to 350 nm were attributed to the absorption of the host. In accordance with previous studies [6,31,32,33], the obtained absorption lines were well assigned to the typical f–f transitions of Er^3+^ ions from ground state ^4^I_15/2_ to the successive excited states including ^4^G_7/2_, ^4^G_9/2_, ^4^G_11/2_, ^2^H_9/2_, ^4^F_5/2_, ^4^F_7/2_, ^2^H_11/2_, ^4^S_3/2_, ^4^F_9/2_, ^4^I_9/2_, ^4^I_11/2_, and ^4^I_13/2_, among which, ^4^I_15/2_ → ^4^G_11/2_ was the dominant one (see the inset in Figure 4). 

**Figure 5:**
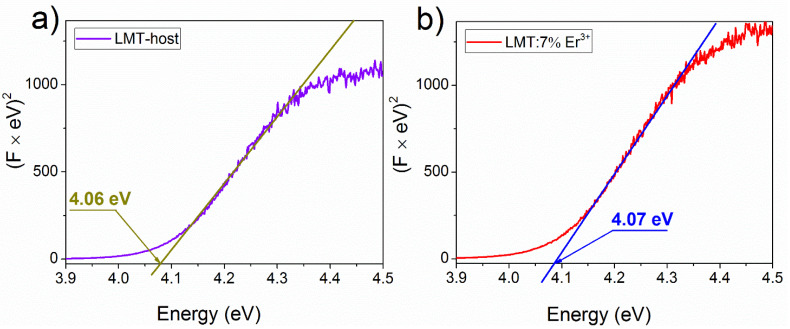
Forbidden energy bandgaps (E_g_) of La_2_MgTiO_6_ host (**a**) and La_2_MgTiO_6_:7% Er^3+^ (**b**) calculated from the absorption spectra obtained from the reflection spectra applying the modified Kubelka–Munk function (also known as the Tauc method).

To determine the energy of the forbidden bandgap of the host and the sample doped with 7% Er^3+^, the modified Kubelka–Munk function (also known as the Tauc method) [34] was applied to the 300 K absorption spectra. The energy gap between the top of the valence band and the bottom of the conduction band of the host and LMT:7% Er^3+^ were found to be 4.06 eV and 4.07 eV, respectively (Figure 5). The insignificant difference between them could have resulted from a measurement error. This value was slightly higher than that (3.98 eV) obtained for the same Pr^3+^-doped host [17].

### 3.3. Luminescence Studies

**Figure 6:**
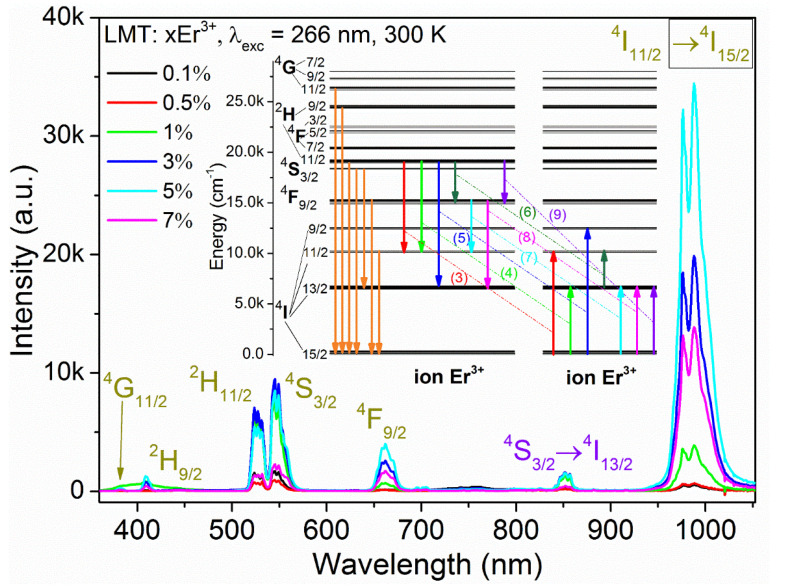
The 300 K emission spectra of La_2_MgTiO_6_:x Er^3+^ (x = 0.1%, 0.5%, 1%, 3%, 5%, or 7%) recorded under a 266 nm excitation Nd:YAG line. Inset: Some possible cross-relaxation processes in La_2_MgTiO_6_:Er^3+^.

A wide spectral range from visible to the near-infrared of LMT doped with different concentrations of Er^3+^ ions, observed for 266 nm wavelength direct excitation of TiO_6_ groups, is shown in Figure 6. As can be seen, there was no difference in the shape of the emission spectrum across the samples. According to the absorption spectrum (Figure 4) and the emission spectra (Figure 6), the assignment of all energy levels of Er^3+^ ions was tabulated in Table S1.

Similarly to the LMT samples doped with Eu^3+^ [3] and Nd^3+^ [2], a broad emission band of TiO_6_ groups also appeared from 400 nm to 550 nm at 77 K (Figure S2). The energy transfer from TiO_6_ groups to Er^3+^ ions was perfectly exemplified by temperature and Er^3+^ concentration. A higher Er^3+^ content resulted in a lower intensity of the emission of TiO_6_ groups. This process was also facilitated by elevating temperature. No TiO_6_ emission was observed at room temperature (Figure 6). For visible emission including ^2^H_11/2_, ^4^S_3/2_, and ^4^F_9/2_ to the ground state, ^4^I_15/2_ located in the green, yellow, and red regions was centered at 531.7 nm (18,807 cm^−1^), 549 nm (18,215 cm^−1^), and 662 nm (15,106 cm^−1^), respectively. In addition, infrared emission from ^4^I_11/2_ and ^4^I_13/2_ levels to the ground state was maximum at 988 nm (10,121 cm^−1^) and 1524 nm (6562 cm^−1^), respectively (Figure S3). Furthermore, the emission spectra were also contributed to by a few less intense transitions corresponding to ^4^G_11/2_, ^2^H_9/2_ → ^4^I_15/2_, and ^4^S_3/2_ → ^4^I_13/2_ levels.

The energy difference (∆E) between ^2^H_11/2_ and ^4^S_3/2_ was 592 cm^−1^; this value is similar to that found in LaAlO_3_:Er^3+^ with 660 cm^−1^ [6]. Due to the small energy difference, the transition from ^2^H_11/2_ to ground state can be easily populated at room temperature by thermalization (see Figure 6).

Temperature plays an important role in the thermal population of ^2^H_11/2_ from the ^4^S_3/2_ level. It is regulated by the following Boltzmann distribution formula:

(1) $$\frac{N_{A}}{N_{B}}=\frac{g_{A}}{g_{B}}\exp\left(\frac{-\Delta E}{\mathrm{kT}}\right),$$

where N_A_ (N_B_) is the population of level ^2^H_11/2_ (^4^S_3/2_), g_A_ (g_B_) is the degeneration of level ^2^H_11/2_ (^4^S_3/2_), k is the Boltzmann constant, and ΔE is the energy difference between these levels. These levels were further considered in terms of temperature sensing.

Moreover, the integrated emission intensity of all samples is plotted in the inset. It can be clearly seen that the integrated intensity moderately increased with Er^3+^ concentration. The highest intensity was achieved for the sample doped with 5% Er^3+^ (Figure S4). Above this threshold value, the intensity decreased as a result of concentration quenching. This phenomenon could result from an exchange interaction or multipolar interaction depending on the critical transfer distance R_c_ between Er^3+^ ions. Therefore, to better understand, Blasse’s model was applied [35].

(2) $$R_{C}=2\times {\left(\frac{3V}{4{\pi x}_{C}N}\right)}^{1/3},$$

where V is the unit cell volume, and x_c_ is the quenching concentration. For samples doped with 5% Er^3+^, V = 242.45 Å^3^, x_c_ = 0.05, and N = 4. The value of $R_{C}$ was equal to 13.2 Å, indicating that the quenching mechanism which occurred in LMT:Er^3+^ samples was predominantly regulated by the electric multipolar interaction. A similar phenomenon was previously observed in LMT doped with Nd^3+^ ions [2].

The analysis of the emission spectra of LMT:Er^3+^ allowed concluding that, with the increase in the concentration of doping ions, two competing phenomena are observed. The increased amount of Er^3+^ ions initially led to an increase in the emission intensity of all bands (see Figure S5); however, the intensity of each of these bands changed differently depending on the Er^3+^ concentration. Due to thermalization at 300 K, both ^2^H_11/2_ and ^4^S_3/2_ levels could be regarded as one. It can be seen that, in the range 1–5% Er^3+^, the emission intensity of ^4^S_3/2_ remained almost constant with a local maximum for 3%. On the other hand, the emission of the ^4^F_9/2_ and ^4^I_11/2_ levels increased from the 0.5% Er^3+^ concentration. This was mainly to the detriment of the (^2^H_11/2_, ^4^S_3/2_) level, which transferred energies to these lower levels in the process of cross-relaxation. As the dopant concentration increased, the likelihood of these processes increased as a function of the number of Er^3+^ ion pairs. If one of the ions in a pair was excited and the other was not, the first would be the donor and the second would be the acceptor, and the energy transfer processes could be described as follows:

(^2^H_11/2_ + ^4^S_3/2_, ^4^I_15/2_) → (^4^I_11/2_, ^4^I_11/2_), ΔE = −1130 cm^−1^, (3)

(^2^H_11/2_ + ^4^S_3/2_, ^4^I_15/2_) → (^4^I_11/2_, ^4^I_13/2_), ΔE = 2210 cm^−1^, (4)

(^2^H_11/2_ + ^4^S_3/2_, ^4^I_15/2_) → (^4^I_13/2_, ^4^I_9/2_), ΔE = 93 cm^−1^. (5)

At continuous wave excitation, the ^4^I_13/2_ level would be populated; therefore, it is possible to write another path of the CR process as follows:

(^2^H_11/2_ + ^4^S_3/2_, ^4^I_13/2_) → (^4^I_9/2_, ^4^I_11/2_), ΔE = 436 cm^−1^. (6)

The ^4^I_11/2_ level could also be fed in another cross-relaxation process from the ^4^F_9/2_ level as follows:

(^4^F_9/2_, ^4^I_15/2_) → (^4^I_11/2_, ^4^I_13/2_) ΔE = −1530 cm^−1^. (7)

The ^4^F_9/2_ level could also be drained by the following process:

(^4^F_9/2_, ^4^I_15/2_) → (^4^I_13/2_, ^4^I_13/2_) ΔE = 1500 cm^−1^. (8)

There was a certain probability of the following CR populating the 4F_9/2_ level:

(^2^H_11/2_ + ^4^S_3/2_, ^4^I_15/2_) → (^4^F_9/2_, ^4^I_13/2_) ΔE = −2280 cm^−1^. (9)

Please note that the maximum energy phonon of this host was found to be 741 cm^−1^ [36]. In the processes described by Equations (3), (7), and (9), there is a shortage of energy, which can easily be obtained from the crystal lattice at 300 K. These are phonon-assisted processes; however, the process described by Equation (9), requiring the assistance of up to three phonons, is the least likely in this list. In the processes described by Equations (4) and (8), a small excess of energy would be easily absorbed by the crystal lattice. All processes are pictured in the inset of Figure 6.

### 3.4. Luminescent Decay Profiles

To better understand the quenching behavior of the green, red, and IR emission, the decay profiles of some samples at room temperature, registered at 549 nm, 662 nm, and 988 nm corresponding to ^4^S_3/2_, ^4^F_9/2_, and ^4^I_11/2_ levels were taken into account (Figure S6). Nonexponential decay was observed for the samples doped with more than 1% Er^3+^. Due to the thermalization phenomenon, the decay of the ^4^S_3/2_ level consisted of two components attributed to ^4^S_3/2_ and ^2^H_11/2_, respectively. Similarly, nonexponential luminescent decay monitored at ^4^F_9/2_ was contributed by the ^4^F_9/2_ and ^4^S_3/2_ levels. The decay time of ^4^F_9/2_ level was twofold shorter than that of the ^4^S_3/2_ level observed for the samples containing less than 3% Er^3+^. Among these levels, the decay time of the ^4^I_11/2_ level was the longest one, as also observed for LaAlO_3_:Er^3+^ [6]. In addition, a very fast rise time was obtained for all samples as a result of cross-relaxation processes (Figure S6 and Table S2).

Figure S5 shows the integrated emission intensity of some levels, including ^2^H_11/2_ (I_2_), ^4^S_3/2_ (I_3_), ^4^F_9/2_ (I_4_), and ^4^I_11/2_ (I_6_) to ground state ^4^I_15/2_. The observed emission of each level can be contributed by radiative emission or nonradiative relaxation (multi-phonon relaxation) and cross-relaxation phenomena. 

**Figure 7:**
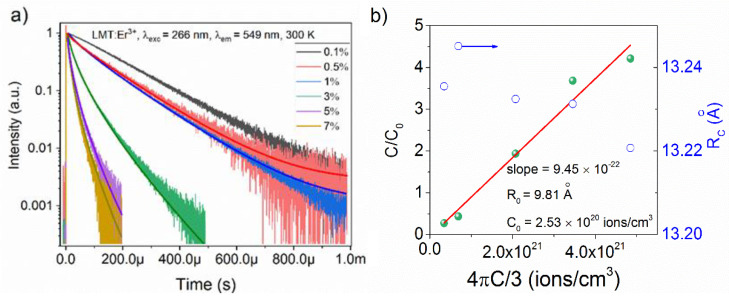
(**a**) The 300 K decay profiles under 266 nm excitation, monitored at 549 nm and fitted to the Inokuti–Hirayama function (solid lines) for S = 6; (**b**) critical transfer distance $R_{0}$ and critical concentration $C_{0}$ calculated from the slope of the plot of $\left(C/C_{0}\right)$ vs. (4πC/3) and the interionic distance between dopants (R_c_) of all Er^3+^-doped samples (right *Y*-axis).

As can be clearly seen, the 300 K decay profiles upon 266 nm excitation (except for the sample doped with 0.1% Er^3+^) exhibited a nonexponential behavior on account of the energy transfer between Er^3+^ ions (Figure 7). Thus, the Inokuti–Hirayama model was used to calculate the critical transfer distance $R_{0}$ and the critical concentration $C_{0}$ by the following equation [37]:

(10) $$I_{t}=I_{0}\exp^{\left[\frac{-t}{\tau }-\alpha \times {\left(\frac{t}{\tau }\right)}^{\frac{3}{s}}\right]}+\left(\mathrm{offset}\right),$$

where $I_{0},{\;I}_{t}$ represent the emission intensity at t = 0 and after pulse excitation, τ is the radiative decay time, C is the Er^3+^ concentration, $C_{0}$ is the Er^3+^ critical concentration, $\Gamma \left(1-\frac{3}{S}\right)$ is the gamma function, S defines the dipole–dipole, dipole–quadrupole, and quadrupole–quadrupole interactions corresponding to S = 6, 8, and 10, respectively, and Q is the energy transfer parameter [37,38,39,40].

(11) $$\alpha =\frac{4\pi }{3}\times \Gamma \left(1-\frac{3}{S}\right)\times C\times R_{0}^{3},$$

where $R_{0}$ stands for the critical transfer distance at which the energy transfer efficiency achieves 50% and is equal the radiative decay rate $\tau_{0}^{-1}$.

The correlation of $C,{\;C}_{0},{\;R}_{0}\;$can be derived from Equations (10) and (11) to yield the following formula [37,38,39,40]:

(12) $$\frac{C}{C_{0}}=\frac{4{\pi \mathrm{CR}}_{0}^{3}}{3}.$$

The donor–acceptor energy transfer parameter $\left(C_{\mathrm{da}}\right)$ and the energy transfer probability $\left(W_{\mathrm{da}}\right)$ can be calculated using the following equations [40,41,42,43]:

(13) $$C_{\mathrm{da}}=R_{0}^{6}\times \tau_{0}^{-1},$$

(14) $$W_{\mathrm{da}}=R_{0}^{-6}\times C_{\mathrm{da}}={\;\tau }_{0}^{-1}.$$

The energy transfer decay rates of the samples as a function of Er^3+^ concentration can be estimated as follows:

(15) $$W_{\mathrm{tot}}=W_{\mathrm{rad}}+W_{\mathrm{da}}=\frac{1}{\tau_{\mathrm{rad}}}+W_{\mathrm{da}},$$

where $\tau_{\mathrm{rad}}$ is supposedly equal to the decay time of the sample doped with 0.1% Er^3+^ ($\tau_{\mathrm{rad}\;}$= 166 μs, Table S2).

(16) $$W_{\mathrm{tot}}={\mathrm{aC}}^{n}+b,$$

where n indicates the strength of the multipole interaction between dopants; a weak interaction is exhibited by n = 1, while a strong one is exhibited by n = 2 [43].

As seen in Figure 7, the dipole–dipole interaction was responsible for the energy transfer in all samples, as exemplified by the best-fitting curves for S = 6. The experimental data can be described by Equation (12) as a linear function of the density of Er^3+^ ions (Figure 7b). The value of $R_{0}$ was 9.81 Å, similar to the $R_{c}$ derived from the Blasse model. However, it is worth emphasizing that the values have different physical meaning [44]. Furthermore, $C_{0}$ = $2.53\times 10^{20}$ ions/cm^−3^, whereas $C_{\mathrm{da}}\;$and $W_{\mathrm{da}}$ were estimated by considering the radiative decay time value of the sample doped with 0.1% Er^3+^ as $\tau_{\mathrm{rad}}$ = 166 μs. Thereafter, $C_{\mathrm{da}}\;$and $W_{\mathrm{da}}$ were found to be $5.38\times 10^{-39}\;$cm^6^·s^−1^ and 6020 s^−1^, respectively. With n = 1.8 obtained from the fitting curve (Figure S7), one can conclude that a strong interaction among Er^3+^ ions happened in LMT at 300 K.

### 3.5. Temperature-Sensing Ability

**Figure 8:**
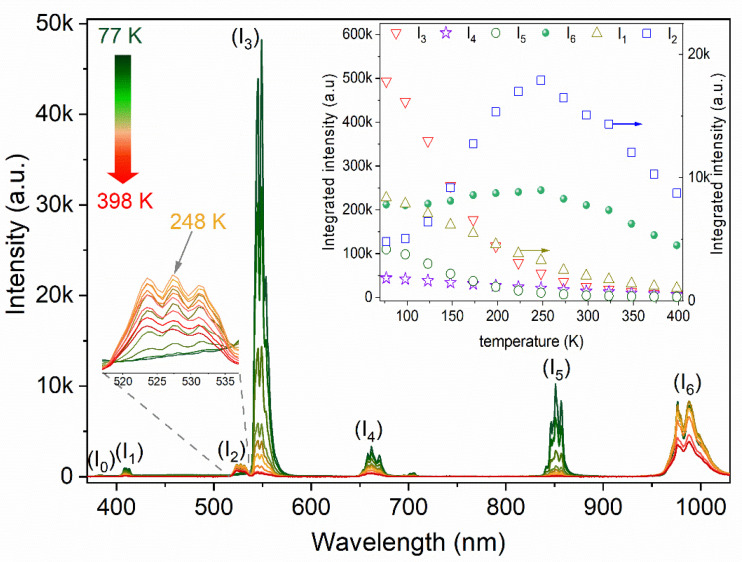
Temperature-dependent emission spectra of La_2_MgTiO_6_:5% Er^3+^ excited at 266 nm in the range 77–398 K. Inset: Integrated emission intensity for distinct transitions as a function of temperature.

Furthermore, the temperature dependence of the emission intensity of LMT 5% Er^3+^ is presented in Figure 8 for the 77–398 K range, and the temperature-dependent integrated emission intensity of some levels is also shown in the inset. It is shown that the most intense emission came from ^4^S_3/2_ → ^4^I_15/2_ (I_3_). In general, I_3_ and some other levels, including ^2^H_9/2_ → ^4^I_15/2_ (I_1_), ^4^S_3/2_ → ^4^I_13/2_ (I_5_), and ^4^F_3/2_ → ^4^I_15/2_ (I_4_), are strongly depopulated by elevating temperature, especially I_3_ since it acts as an energy pump for the ^2^H_11/2_ x2192; ^4^I_15/2_ transition (I_2_) via the thermalization process. The emission of I_2_ reached its maximum at 248 K, before being quenched very quickly at a higher temperature. On the other hand, the ^4^I_11/2_ → ^4^I_15/2_ transition (I_6_) was quite stable, remaining mostly unchanged until 248 K, at which point it then exhibited a significant decrease.

**Figure 9:**
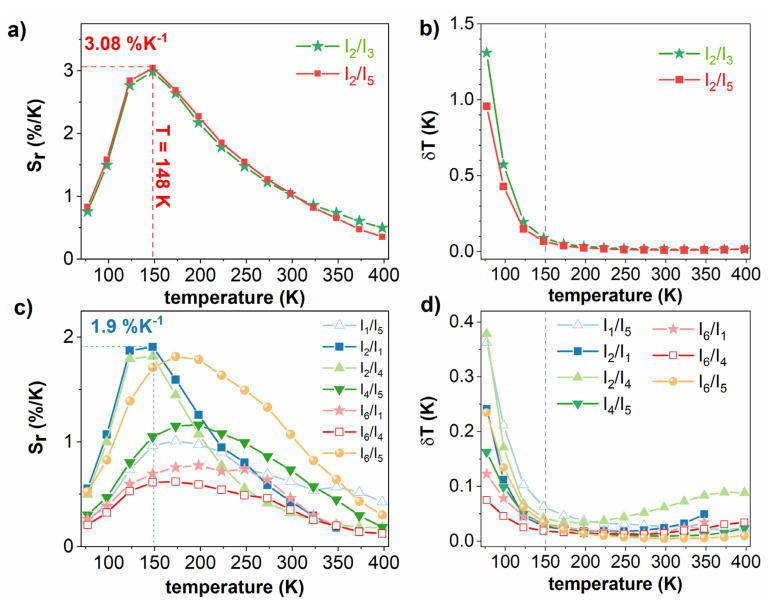
Relative sensitivities (S_r_) and temperature uncertainties (δT) derived from thermometric parameters based on thermally coupled levels (**a**,**b**) and uncoupled levels (**c**,**d**).

To verify the potential applicability of using LMT:Er^3+^ as a noncontact thermometer, all possible combinations of Er^3+^ emission bands were taken into account in the temperature range of 77–398 K (Figure 9). The first was devoted to the most frequently used, thermally coupled levels of Er^3+^, including ^2^H_11/2_ → ^4^I_15/2_ and ^4^S_3/2_ → ^4^I_15/2_ (abbreviated as I_2_/I_3_) and a combination which also started from the same level ^4^S_3/2_, i.e., ^2^H_11/2_ → ^4^I_15/2_ and ^4^S_3/2_ → ^4^I_13/2_ (I_2_/I_5_). The second was constructed on the basis of uncoupled levels; due to the different thermal behaviors through radiative emission, it is highly probable that these combinations would also be sensitive to temperature fluctuations.

The temperature-sensing performance of a thermometer can be evaluated through the absolute S_a_ and relative sensitivity S_r_ parameters. However, S_a_ depends on the sample characteristics and experimental setup, whereas S_r_ can be used for a comparison among distinct materials [24].

(17) $$\Delta =\frac{I_{i}}{I_{j}},$$

(18) $$S_{a}=\frac{\partial \Delta }{\Delta T},\;$$

(19) $$S_{r}=\frac{1}{\Delta }\left|\frac{\partial \Delta }{\Delta T}\right|,$$

where thermometric parameter Δ is denoted as the ratio between two different emission levels I_i_, I_j_.

For thermally coupled levels, the emission intensity of the ^2^H_11/2_ → ^4^I_15/2_ transition was strongly populated by thermalization via the ^4^S_3/2_ level. As a result, the emission intensity of the transition coming from ^4^S_3/2_ decreased, i.e., ^4^S_3/2_ → ^4^I_15/2_ (I_3_) and ^4^S_3/2_ → ^4^I_13/2_ (I_5_). However, ^4^S_3/2_ → ^4^I_13/2_ exhibited a steady quench with respect to ^4^S_3/2_ → ^4^I_15/2_, resulting in a much faster growth in the thermometric parameter and a higher absolute sensitivity (S_a_) for I_2_/I_5_ (see Figure S8a,b). As shown in Figure 9, the relative sensitivities (S_r_) of given coupled levels were identical as predicted, since both (I_3_) and (I_5_) originated from the same level ^4^S_3/2_. The highest S_r_ recorded at 148 K with the values of 2.98%·K^−1^ and 3.08%·K^−1^ corresponded to I_2_/I_3_ and I_2_/I_5_, respectively.

Regarding the thermally uncoupled levels, the S_r_ values are plotted in Figure 9c. The best performance for temperature determination was obtained for the I_2_/I_1_ pair with 1.9%·K^−1^ at 148 K.

It is crucial to note that higher relative sensitivity does not imply more reliable results. Therefore, temperature uncertainty (δT), which is usually intentionally ignored, was determined using the below equation [24].

(20) $$\delta T=\frac{1}{S_{r}}\frac{\delta \Delta }{\Delta }.$$

δT is known as the smallest change in temperature which can be detected by the measurement. In the range from 77–150 K, δT was found to be less than 1.3 K and 0.4 K for coupled levels and uncoupled levels, respectively. From 150–400 K, the values were found to be less than 0.1 K in both cases (Figure 9b,d).

Table 1 presents the relative sensitivities of numerous materials doped or co-doped with Er^3+^ ions. The results in this work completely dominate in comparison with other compounds investigated in the literature. For some samples studied on both thermally coupled and uncoupled levels, it can be seen that the thermally coupled levels had higher sensitivity. However, only a few transitions can work as thermally coupled levels, while many uncoupled levels can provide more promising pairs across the different temperature ranges. Furthermore, the introduction of some co-dopants (Y^3+^, La^3+^, Yb^3+^) into the Er^3+^-doped system was not only to enhance the green up-conversion emission of Er^3+^ ions, but also to create more potential pairs to tackle the limitations of the thermally coupled level-based optical thermometer.

## 4. Conclusions

In this study, La_2_MgTiO_6_:Er^3+^ was prepared via the coprecipitation method. All samples crystallized in n orthorhombic structure with the space group *Pbnm* (62). The chemical composition of the representative sample was in agreement with the theoretical chemical composition. The morphology of the sample was inhomogeneous, and the grain size was distributed in the micro range, from 0.5 to 1 µm. The energy transfer among Er^3+^ ions occurred predominantly via dipole–dipole interactions. The critical transfer distance R_0_, critical concentration C_0_, energy transfer parameter C_da_, and energy transfer probability W_da_ were found to be 9.81 Å, $2.53\times 10^{20}$ ions·cm^−3^, $5.38\times 10^{-39}\;$cm^6^·s^−1^_,_ and 6020 s^−1^, respectively. More importantly, the low-temperature-sensing ability of La_2_MgTiO_6_:5% Er^3+^ was explored using the ratio of emission intensity of the thermally coupled and uncoupled energy levels. The thermally coupled energy levels ^2^H_11/2_ → ^4^I_15/2_ and ^4^S_3/2_ → ^4^I_15/2_ exhibited the greatest sensitivity of around 2.98%·K^−1^ at 148 K, and the uncertainty temperature was less than 1 K in the range of 77–398 K. The abovementioned findings indicate the huge potential of La_2_MgTiO_6_:Er^3+^ for temperature-sensing operating at low temperature.

## Figures and Tables

**Table 1:** The maximum relative sensitivity S_m_ (%·K^−1^) of some materials doped with Er^3+^ corresponding to thermally coupled level and uncoupled levels at temperature T_m_ (K) with temperature uncertainty δT_m_ (K).

Material	λ_exc_ (nm)	Temperature Range (K)	S_m_ (%·K^−1^)	T_m_ (K)	δT_m_ (K)	References
Al_2_O_3_:Er^3+^, Y^3+^	978	295–973	0.35	450	0.3	[45]
Sr_2_CaWO_6_:Er^3+^, La^3+^	980	250–2000	0.5	439	-	[46]
NaNbO_3_:Er^3+^	976	293–353	0.51	355	-	[47]
NaLaMgWO_6_:Er^3+^	378	303–483	1.04	303	-	[48]
Gd_2_O_3_:Er^3+^, Yb^3+^	980	300–550	1.18	300	-	[49]
Y_2_O_3_:Er^3+^ Coupled levels	379	296–500	1.54	483	-	[18]
Uncoupled levels	379	303–434	0.87	434	-	[18]
LiLaMgWO_6_:Er^3+^	378	303–483	2.24	483	-	[29]
Y_3_Ga_5_O_12_:Er^3+^	488	300-850	0.64	547	-	[50]
La_2_MgTiO_6_:Er^3+^	380	303–483	1.107	303	-	[25]
(solid-state)						
La_2_MgTiO_6_:Er^3+^	266	77–398	2.98	148	0.09	This work
(coprecipitation)						
Coupled levels						
Uncoupled levels	266	77–398	1.9	148	0.027	This work

The obtained result is also easily comparable with various dopants, such as LMT: Pr^3+^ S_r_ = 1.28%·K^−1^ at 350 K [17], LMT:Eu^3+^ S_r_ = 3%·K^−1^ at 77 K [3], LMT:Nd^3+^ S_r_ = 0.83%·K^−1^ at 275 K [2], and LMT:V^5+^ and Cr^3+^ S_r_ = 1.96%·K^−1^ at 165 K [1]. Such an exceptional value of S_r_ of 2.98%·K^−1^ at 148 K demonstrates LMT:5% Er^3+^ as a potential candidate for temperature readout at low temperature.

## Data Availability

Data is contained within the article or supplementary material.

## Supplementary Materials

The following are available online at https://www.mdpi.com/article/10.3390/ma14195557/s1, Figure S1: Lattice parameters (a: black squares, b: blue triangles, c: red circles) changes of La_2_MgTiO_6_:x Er^3+^, (x = 1, 3, 5, 7%) as a function of Er^3+^ concentration. Figure S2: 77 K emission spectra of La_2_MgTiO_6_:x Er^3+^, (x = 0.1, 0.5, 1, 3, 5, 7%) recorded under 266 nm excitation. Figure S3: Emission spectra of La_2_MgTiO_6_:3% Er^3+^ obtained in the infrared regions under 808 excitation. Figure S4: Integrated emission intensity of La_2_MgTiO_6_:x Er^3+^, (x = 0.1, 0.5, 1, 3, 5, 7%) recorded under 266 nm excitation at 300 K. Figure S5: Integrated emission intensity of each level as a function of Er^3+^ concentration at 300 K. Figure S6: 300 K decay profiles of La_2_MgTiO_6_:Er^3+^ and rise time (in the inset) excited at 266 nm and monitored at 549 nm (a), at 662 nm (b), at 988 nm (c). Figure S7: Energy transfer rates Wtot = aCn at 300 K as a function of Er^3+^ concentration. Figure S8. Thermometric parameters (a) and absolute sensitivities, Sa (K-1) (b) based on thermally coupled levels; Thermometric parameters (c) and absolute sensitivities, Sa (K-1) (d) based on thermally uncoupled levels. Table S1: Energy levels of Er^3+^ ions in La_2_MgTiO_6_ double perovskites obtained from the 77 K and 300 K emission spectra and the 300 K absorption spectrum *. Table S2: Decay time of La_2_MgTiO_6_:Er^3+^ obtained at different monitoring wavelength corresponding to 4S3/2 (549 nm), 4F_9/2_ (662 nm), 4I_11/2_ (988 nm) levels and their rise time (*) single-exponential decay for the sample La_2_MgTiO_6_:0.1% Er^3+^.

## References

[1] Stefańska D., Bondzior B., Vu T.H.Q., Grodzicki M., Dereń P.J. (2021). Temperature sensitivity modulation through changing the vanadium concentration in a La_2_MgTiO_6_:V^5+^,Cr^3+^ double perovskite optical thermometer. Dalton Trans..

[2] Vu T., Bondzior B., Stefańska D., Dereń P. (2021). Influence of temperature on near-infrared luminescence, energy transfer mechanism and the temperature sensing ability of La_2_MgTiO_6_:Nd^3+^ double perovskites. Sensors Actuators A Phys..

[3] Bondzior B., Stefańska D., Vũ T., Miniajluk-Gaweł N., Dereń P. (2021). Red luminescence with controlled rise time in La_2_MgTiO_6_:Eu^3+^. J. Alloys Compd..

[4] Antic-Fidancev E., Deren P., Krupa J.-C. (2004). Energy levels and crystal field calculations of Er^3+^ in LaAlO_3_. J. Alloys Compd..

[5] Dereń P., Mahiou R., Pazik R., Lemanski K., Strek W., Boutinaud P. (2008). Upconversion emission in CaTiO_3_:Er^3+^ nanocrystals. J. Lumin..

[6] Deren P., Mahiou R. (2007). Spectroscopic characterisation of LaAlO_3_ crystal doped with Er^3+^ ions. Opt. Mater..

[7] Su B., Xie H., Tan Y., Zhao Y., Yang Q., Zhang S. (2018). Luminescent properties, energy transfer, and thermal stability of double perovskites La_2_MgTiO_6_:Sm^3+^, Eu^3+^. J. Lumin..

[8] Li W., Chen T., Xia W., Yang X., Xiao S. (2018). Near-infrared emission of Yb^3+^ sensitized by Mn^4+^ in La_2_MgTiO_6_. J. Lumin..

[9] Srivastava A., Comanzo H., Brik M. (2018). Luminescence of Bi_3+_ in the double perovskite, La_2_MgTiO_6_. Opt. Mater..

[10] Yin X., Yao J., Wang Y., Zhao C., Huang F. (2012). Novel red phosphor of double perovskite compound La_2_MgTiO_6_:x Eu^3+^. J. Lumin..

[11] Huang J., Qin M., Yu J., Ma A., Yu X., Liu J., Zheng Z., Wang X. (2019). La_2_MgTiO_6_:Eu^2+^/TiO_2_-based composite for methyl orange (MO) decomposition. Appl. Phys. A.

[12] Srivastava A.M., Brik M.G., Comanzo H.A., Beers W.W., Cohen W.E., Pocock T. (2018). Spectroscopy of Mn^4+^ in Double Perovskites, La_2_LiSbO_6_ and La_2_LiSbO_6_: Deep Red Photon Generators for Agriculture LEDs. ECS J. Solid State Sci. Technol..

[13] Li L., Qin F., Zhou Y., Miao J., Zhang Z. (2020). Origin of the giant thermal enhancement of the Er^3+^ ion’s 4I_9/2_-4I_15/2_ photoluminescence. Spectrochim. Acta Part A Mol. Biomol. Spectrosc..

[14] Gai S., Zhu H., Gao P., Zhou C., Kong Z., Molokeev M.S., Qi Z., Zhou Z., Xia M. (2020). Structure analysis, tuning photoluminescence and enhancing thermal stability on Mn^4+^-doped La_2_-xYxMgTiO_6_ red phosphor for agricultural lighting. Ceram. Int..

[15] Takeda Y., Kato H., Kobayashi M., Kobayashi H., Kakihana M. (2015). Photoluminescence Properties of Mn^4+^-activated Perovskite-type Titanates, La_2_MTiO_6_:Mn^4+^(M = Mg and Zn). Chem. Lett..

[16] Xie H., Su B., Tan Y., Zhao Y., Chai S. (2018). Effect of Gd^3+^, Bi^3+^, or Sm^3+^ on luminescent properties of La_2_−x MgTiO_6_:x Eu^3+^ phosphors. Luminescence.

[17] Shi R., Lin L.-T., Dorenbos P., Liang H. (2017). Development of a potential optical thermometric material through photoluminescence of Pr^3+^ in La_2_MgTiO_6_. J. Mater. Chem. C.

[18] Brandão-Silva A.C., Gomes M.A., Novais S.M.V., Macedo Z.S., Avila J.F.M., Rodrigues J.J., Alencar M.A.R.C. (2018). Size influence on temperature sensing of erbium-doped yttrium oxide nanocrystals exploiting thermally coupled and uncoupled levels’ pairs. J. Alloys Compd..

[19] Rijckaert H., Kaczmarek A.M. (2020). Ho^3+^–Yb^3+^ doped NaGdF_4_ nanothermometers emitting in BW-I and BW-II. Insight into the particle growth intermediate steps. Chem. Commun..

[20] Stefańska D., Dereń P.J. (2020). High Efficiency Emission of Eu^2+^ Located in Channel and Mg-Site of Mg_2_Al_4_Si_5_O_18_ Cordierite and Its Potential as a Bi-Functional Phosphor toward Optical Thermometer and White LED Application. Adv. Opt. Mater..

[21] Stefańska D., Stefanski M., Dereń P.J. (2021). Unusual emission generated from Ca_2_Mg_0.5_AlSi_1.5_O_7_:Eu^2+^ and its potential for UV-LEDs and non-contact optical thermometry. J. Alloys Compd..

[22] Trojan-Piegza J., Brites C.D.S., Ramalho J.F.C.B., Wang Z., Zhou G., Wang S., Carlos L.D., Zych E. (2020). La_0.4_Gd_1.6_Zr_2_O_7_:0.1%Pr transparent sintered ceramic—A wide-range luminescence thermometer. J. Mater. Chem. C.

[23] Sójka M., Brites C.D.S., Carlos L.D., Zych E. (2020). Exploiting bandgap engineering to finely control dual-mode Lu_2_(Ge,Si)O_5_:Pr^3+^ luminescence thermometers. J. Mater. Chem. C.

[24] Brites C., Millán A., Carlos L. (2016). Lanthanides in Luminescent Thermometry. Metals.

[25] Hua Y., Yu J.S. (2021). Strong Green Emission of Erbium(III)-Activated La_2_MgTiO_6_ Phosphors for Solid-State Lighting and Optical Temperature Sensors. ACS Sustain. Chem. Eng..

[26] Wade S., Collins S.F., Baxter G. (2003). Fluorescence intensity ratio technique for optical fiber point temperature sensing. J. Appl. Phys..

[27] Ćirić A., Aleksić J., Barudžija T., Antić Ž., Đorđević V., Medić M., Periša J.B., Zeković I., Mitrić M., Dramićanin M.D. (2020). Comparison of Three Ratiometric Temperature Readings from the Er^3+^ Upconversion Emission. Nanomaterials.

[28] Rakov N., Maciel G.S. (2018). Exploring the ^4^I_13/2_ → ^4^I_15/2_ radiative transition from Er^3+^ in Y_2_O_3_ for temperature sensing. J. Lumin..

[29] Ran W., Noh H.M., Park S.H., Lee B.R., Kim J.H., Jeong J.H., Shi J., Liu G. (2019). Simultaneous bifunctional application of solid-state lighting and ratiometric optical thermometer based on double perovskite LiLaMgWO_6_:Er^3+^ thermochromic phosphors. RSC Adv..

[30] Shannon R.D. (1976). Revised effective ionic radii and systematic studies of interatomic distances in halides and chalcogenides. Acta Crystallogr. A.

[31] Ölsä J., Säilynoja E., Lamminmäki R.-J., Deren P., Strek W., Porcher P. (1997). Crystal field energy level scheme of Er^3+^ in GdOCl Parametric analysis. J. Chem. Soc. Faraday Trans..

[32] Stręk W., Dereń P., Maruszewski K., Pawlik E., Wojcik W., Malashkevich G., Gaishun V., Strȩk W. (1998). Spectroscopic properties of erbium doped silica glasses obtained by sol-gel method. J. Alloys Compd..

[33] Macalik L., Dereń P., Hanuza J., Stręk W., Demidovich A., Kuzmin A. (1998). Effect of random distribution and molecular interactions on optical properties of Er^3+^ dopant in KY(WO_4_)_2_ and Ho^3+^ in KYb(WO_4_)_2_. J. Mol. Struct..

[34] López R., Gómez R. (2012). Band-gap energy estimation from diffuse reflectance measurements on sol–gel and commercial TiO_2_: A comparative study. J. Sol-Gel Sci. Technol..

[35] Blasse G. (1968). Energy transfer in oxidic phosphors. Phys. Lett. A.

[36] Levin I., Vanderah T.A., Amos T.G., Maslar J.E. (2005). Structural Behavior and Raman Spectra of Perovskite-Like Solid Solutions (1 − *x*)LaMg_0.5_Ti _0.5_O_3−*x*_La_2/3_TiO_3_. Chem. Mater..

[37] Inokuti M., Hirayama F. (1965). Influence of Energy Transfer by the Exchange Mechanism on Donor Luminescence. J. Chem. Phys..

[38] Puchalska M., Watras A. (2016). A clear effect of charge compensation through Na^+^ co-doping on luminescent properties of new CaGa_4_O_7_:Nd^3+^. J. Alloys Compd..

[39] Puchalska M., Watras A. (2016). A clear effect of charge compensation through Na^+^ co-doping on the luminescence spectra and decay kinetics of Nd^3+^-doped CaAl_4_O_7_. J. Solid State Chem..

[40] Rudnicka D., Dereń P. (2013). Preliminary spectroscopic properties of K_4_SrSi_3_O_9_ doped with Eu^3+^. Opt. Mater..

[41] Zheng Y., Chen B., Zhong H., Sun J., Cheng L., Li X., Zhang J., Tian Y., Lu W., Wan J. (2011). Optical Transition, Excitation State Absorption, and Energy Transfer Study of Er^3+^, Nd^3+^ Single-Doped, and Er^3+^/Nd^3+^ Codoped Tellurite Glasses for Mid-Infrared Laser Applications. J. Am. Ceram. Soc..

[42] Llanos J., Espinoza D., Castillo R. (2017). Energy transfer in single phase Eu^3+^-doped Y_2_WO_6_ phosphors. RSC Adv..

[43] Bondzior B., Stefanska D., Kubiak A., Dereń P. (2015). Spectroscopic properties of K_4_SrSi_3_O_9_ doped with Sm^3+^. J. Lumin..

[44] Bin Im W., Fellows N.N., Denbaars S., Seshadri R., Kim Y.-I. (2009). LaSr_2_AlO_5_, a Versatile Host Compound for Ce^3+^-Based Yellow Phosphors: Structural Tuning of Optical Properties and Use in Solid-State White Lighting. Chem. Mater..

[45] Dong B., Wang X.J., Li C.R., Liu D.P. (2008). Er^3+^-Y^3+^-Codoped Al_2_O_3_ for High-Temperature Sensing. IEEE Photon-Technol. Lett..

[46] Wang Y., Liu Y., Shen J., Wang X., Yan X. (2018). Controlling optical temperature behaviors of Er^3+^ doped Sr_2_CaWO_6_ through doping and changing excitation powers. Opt. Mater. Express.

[47] Kumar K.U., Santos W.Q., Silva W.F., Jacinto C. (2013). Two photon thermal sensing in Er^3+^/Yb^3+^ Co-doped nanocrystalline NaNbO_3_. J. Nanosci. Nanotechnol..

[48] Ran W., Noh H.M., Park S.H., Lee B.R., Kim J.H., Jeong J.H., Shi J. (2019). Er^3+^-Activated NaLaMgWO_6_ double perovskite phosphors and their bifunctional application in solid-state lighting and non-contact optical thermometry. Dalton Trans..

[49] Šević D., Rabasovic M., Križan J., Savić-Šević S., Marinkovic B.P., Nikolic M. (2020). Effects of temperature on luminescent properties of Gd_2_O_3_:Er, Yb nanophosphor. Opt. Quantum Electron..

[50] León-Luis S.F., Monteseguro V., Rodriguez-Mendoza U., Rathaiah M., Venkatramu V., Lozano-Gorrin A., Valiente R., Munoz A., Lavin V. (2014). Optical nanothermometer based on the calibration of the Stokes and upconverted green emissions of Er^3+^ ions in Y_3_Ga_5_O_12_ nano-garnets. RSC Adv..

