# Supplementary material for: Exploration of the Temperature Sensing Ability of La2MgTiO6:Er3+ Double Perovskites Using Thermally Coupled and Uncoupled Energy Levels
Source: Materials (Basel). 2021 Sep 24;14(19):5557. doi: 10.3390/ma14195557 (PMC8509155; doi:10.3390/ma14195557)
Supplement: Supplementary file 1 [file materials-14-05557-s001.zip › materials-1362977-supplementary.pdf]

materials

Supplementary Materials
Exploration of the Temperature Sensing Ability of $La_2MgTiO_6:Er^{3+}$ Double Perovskites Using Thermally Coupled and Uncoupled Energy Levels

Thi Hong Quan Vu, Bartosz Bondzior, Dagmara Stefańska and Przemysław J. Dereń *

Institute of Low Temperature and Structure Research, Polish Academy of Sciences, Okólna2, 50-422 Wrocław, Poland; q.vu@intibs.pl (T.H.Q.V.); b.bondzior@intibs.pl (B.B.); d.stefanska@intibs.pl (D.S.)
* Correspondence: p.deren@intibs.pl

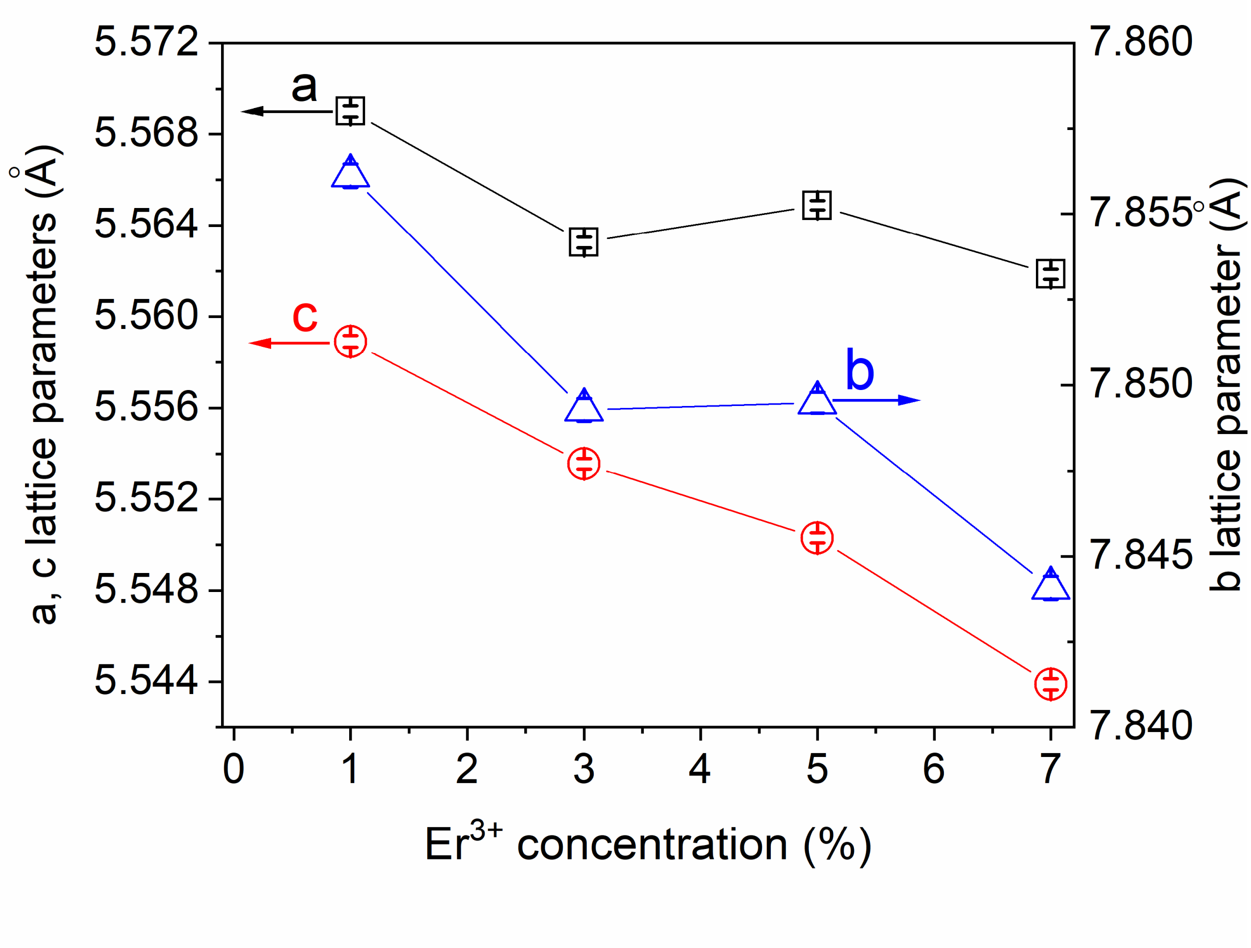

Figure S1. Lattice parameters (a: black squares, b: blue triangles, c: red circles) changes of La_2MgTiO_6: x Er^{3+}, (x = 1, 3, 5, 7 %) as a function of Er^{3+} concentration.

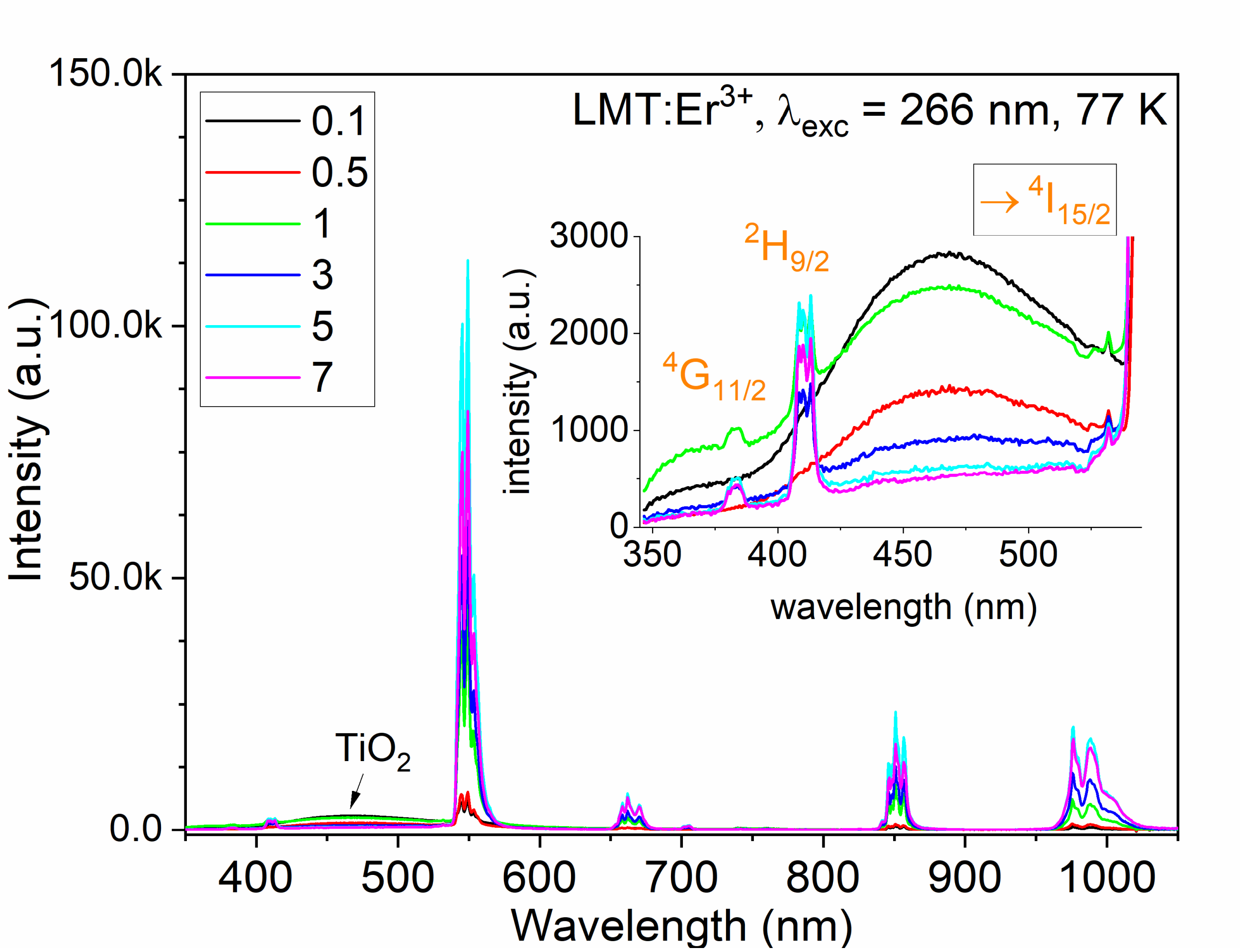

Figure S2. 77 K emission spectra of La_2MgTiO_6: x Er^{3+}, (x = 0.1, 0.5, 1, 3, 5, 7 %) recorded under 266 nm excitation.

Table S1. Energy levels of Er^{3+} ions in La_2MgTiO_6 double perovskites obtained from the 77 K and 300 K emission spectra and the 300 K absorption spectrum*.

Level	Number of levels		Energies (cm^{-1})	ΔE (cm^{-1})
	Experiment	Theory		
$^4I_{15/2}$	6	8	0, 96, 127, 139, 272, 362	362
$^4I_{13/2}$	7	7	6460*, 6527, 6555, 6565*, 6592*, 6666, 6770*	309

$^4I_{11/2}$	2	6	10111*, 10242	131
$^4I_{9/2}$	2	5	12424*, 12563*	139
$^4F_{9/2}$	5	5	14916*, 15099*, 15195, 15230*, 15300*	384
$^4S_{3/2}$	2	2	18335, 18437*	102
$^2H_{11/2}$	6	6	18965, 19066*, 19109*, 19153*, 19190*, 19246*	281
$^4F_{7/2}$	4	4	20325*, 20408*, 20450*, 20521*	196
$^4F_{5/2}$	3	3	21930*, 22026*, 22173*	243
$^4F_{3/2}$	2	2	22447*, 22573*	126
$^2H_{9/2}$	5	5	24361*, 24438*, 24480, 24570*, 24631*	270
$^4G_{11/2}$	1	6	26178*, 26316*, 26385, 26469*	291
$^4G_{9/2}$	2	5	27211*, 27322*	111
$^4G_{7/2}$	1	4	28011*	

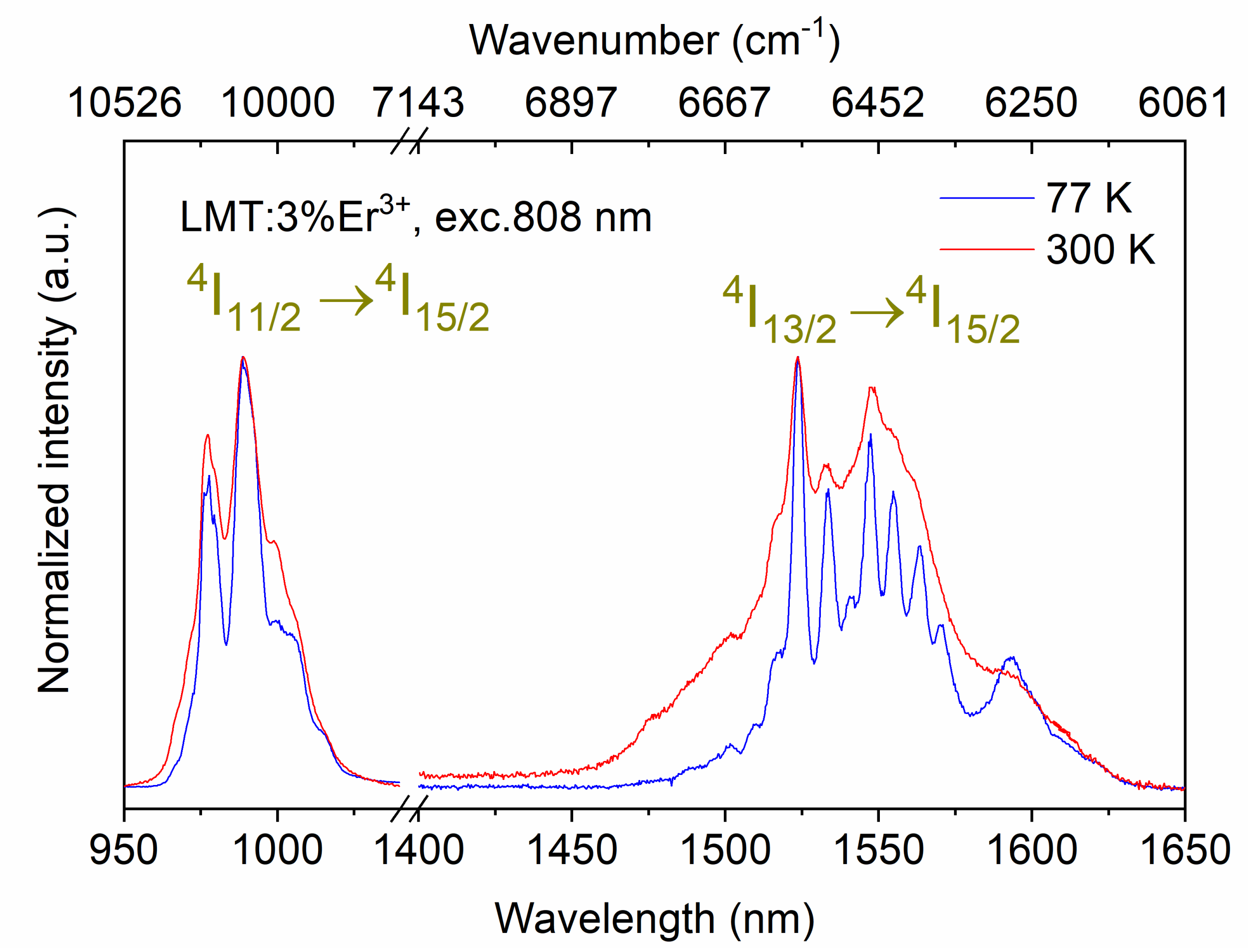

Figure S3. Emission spectra of La_2MgTiO_6: 3 % Er^{3+} obtained in the infrared regions under 808 excitation.

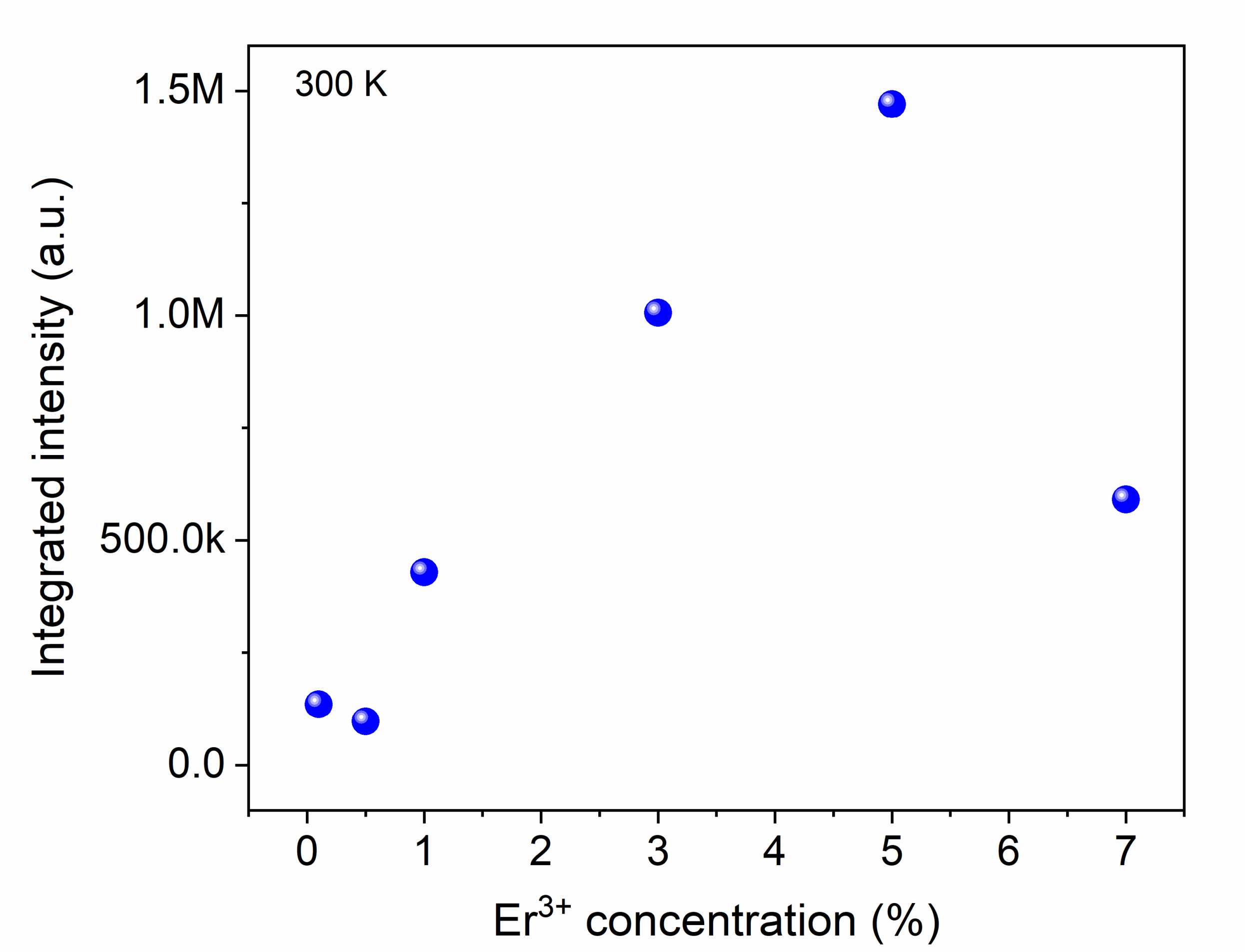

Figure S4. Integrated emission intensity of La_2MgTiO_6: x Er^{3+}, (x = 0.1, 0.5, 1, 3, 5, 7 %) recorded under 266 nm excitation at 300 K.

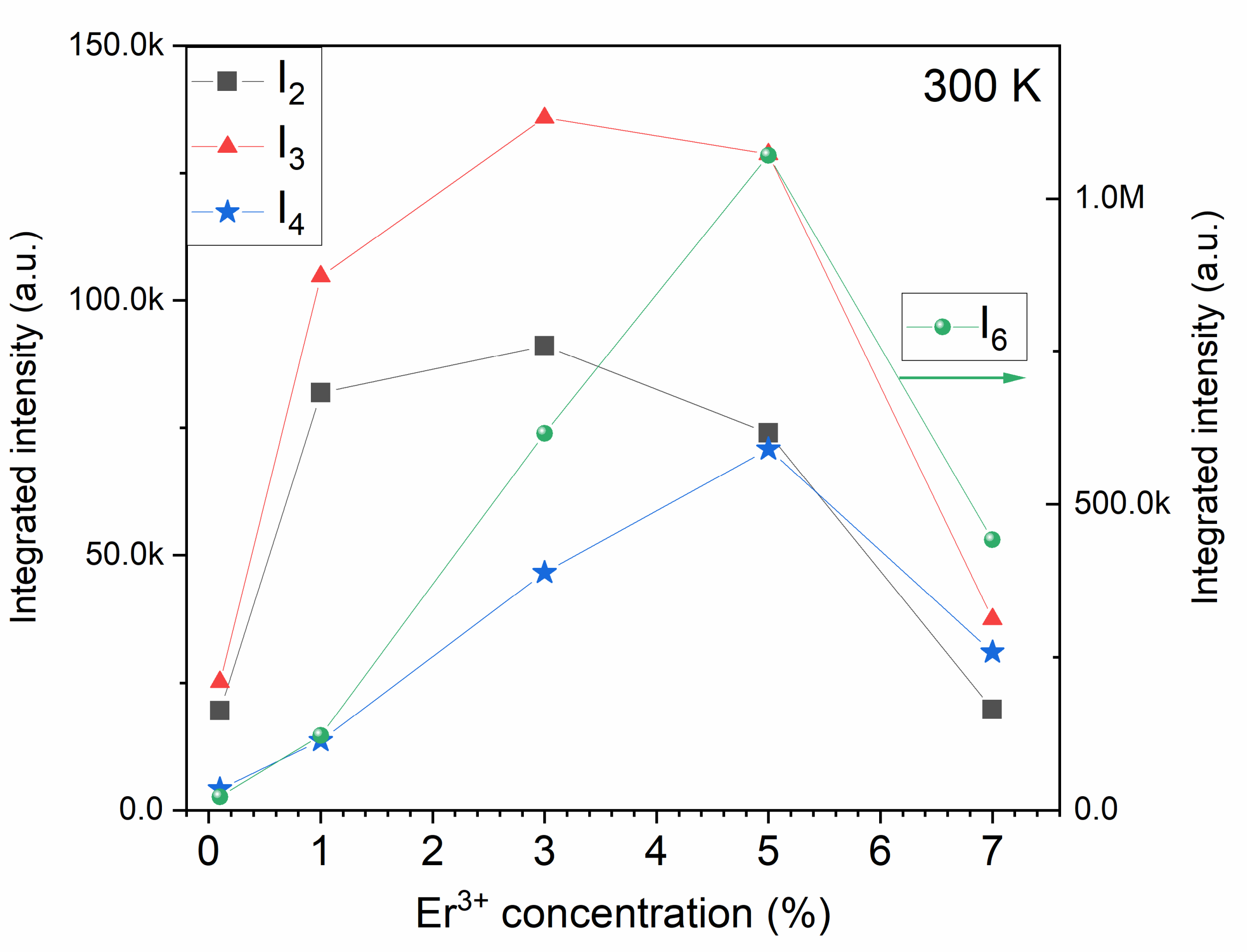

Figure S5. Integrated emission intensity of each level as a function of Er^{3+} concentration at 300 K.

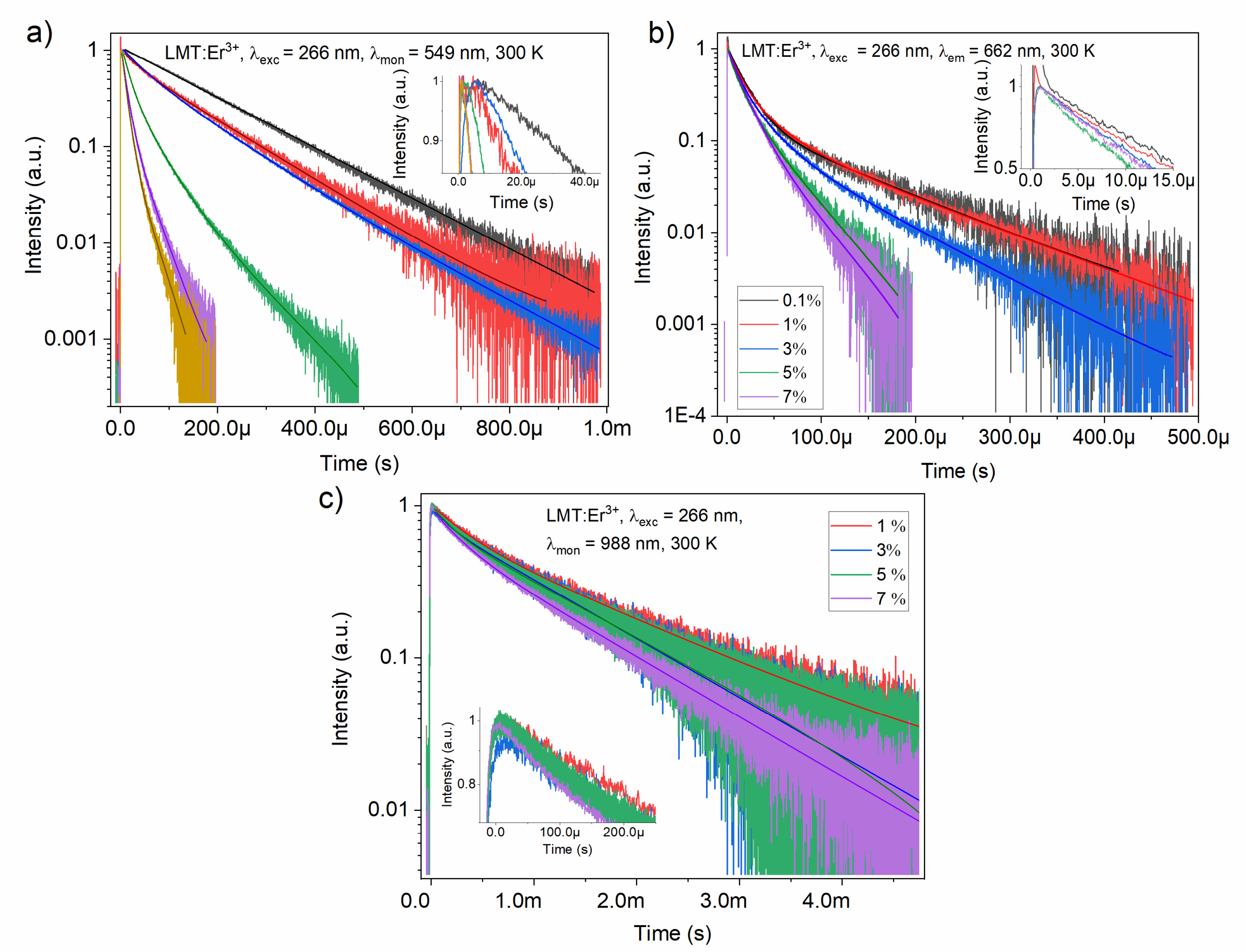

Figure S6. 300 K decay profiles of La_2MgTiO_6: Er^{3+} and rise time (in the inset) excited at 266 nm and monitored at 549 nm (a), at 662 nm (b), at 988 nm (c).

The 300 K decay profiles of all samples exhibited multiexponential functions determined as the following equation:

$$y = A_1 \times e^{-x/\tau_1} + A_2 \times e^{-x/\tau_2} + A_3 \times e^{-x/\tau_3} \quad (1)$$

where τ_1, τ_2, τ_3 are decay time constants, and A_1, A_2, A_3 are the amplitudes.

The amplitude average lifetime was calculated using the below equation [1]:

$$\tau_{avg} = \frac{A_1 \times \tau_1^2 + A_2 \times \tau_2^2 + A_3 \times \tau_3^2}{A_1 \times \tau_1 + A_2 \times \tau_2 + A_3 \times \tau_3}$$

Table S2. Decay time of La_2MgTiO_6: Er^{3+} obtained at different monitoring wavelength corresponding to $^4S_{3/2}$ (549 nm), $^4F_{9/2}$ (662 nm), $^4I_{11/2}$ (988 nm) levels and their rise time (*) single-exponential decay for the sample La_2MgTiO_6: 0.1% Er^{3+}.

	**λ_{em} = 549 nm**		**λ_{em} = 662 nm**		**λ_{em} = 988 nm**	
Er^{3+} (%)	τ_{avg} (μs)	τ_{rise} (μs)	τ_{avg} (μs)	τ_{rise} (μs)	τ_{avg} (μs)	τ_{rise} (μs)
0.1	166*	1.94	66.7	---	---	---
1	124	1.58	66.7	---	1330	4.39
3	44.1	0.185	43.4	0.15	1090	5.9
5	18.8	0.16	26.4	0.15	1150	4.73
7	14.4	0.13	23.4	0.16	1010	3.64

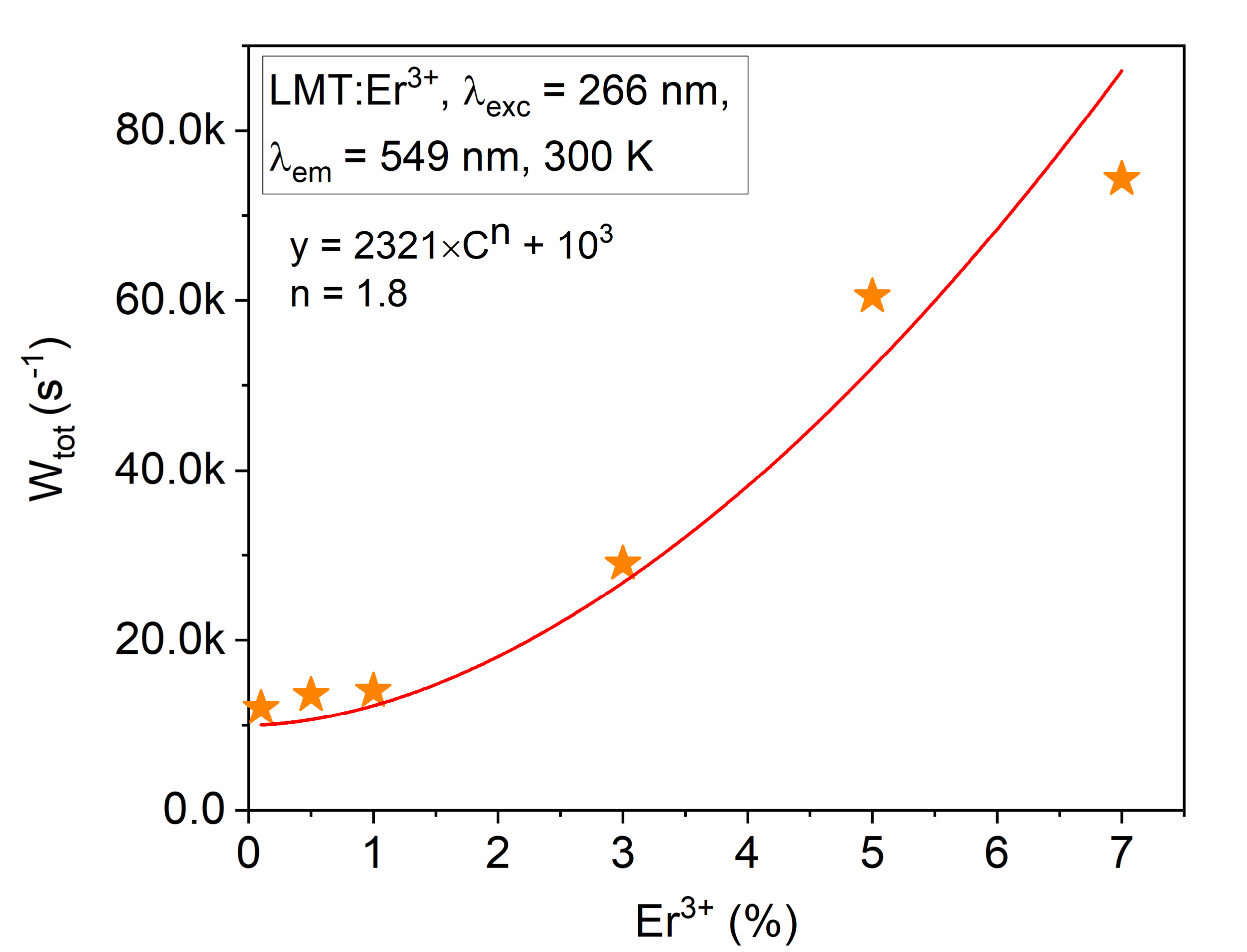

Figure S7. Energy transfer rates $W_{tot} = aC^n$ at 300 K as a function of Er^{3+} concentration.

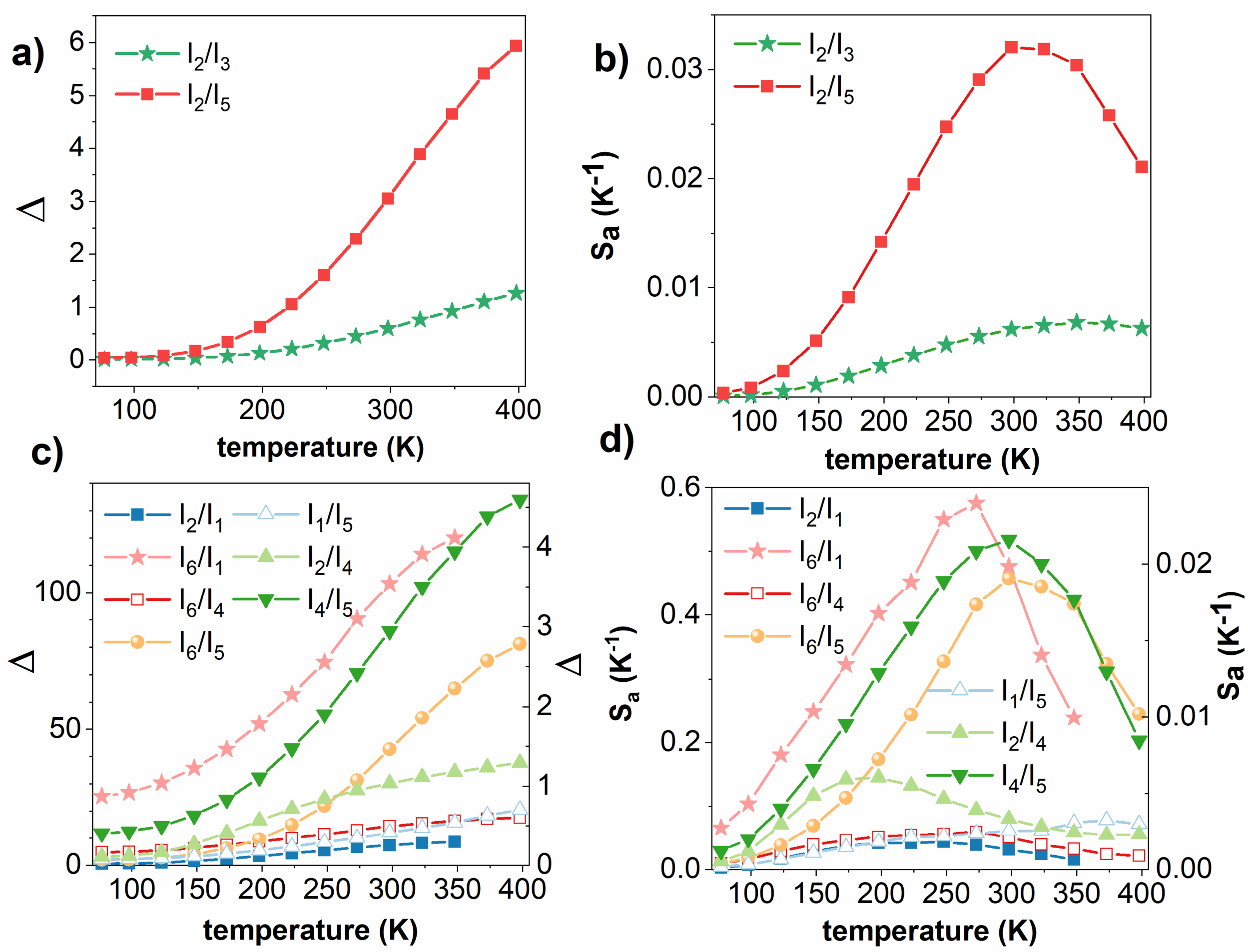

Figure S8. Thermometric parameters (a) and absolute sensitivities, S_a (K^{-1}) (b) based on thermally coupled levels; Thermometric parameters (c) and absolute sensitivities, S_a (K^{-1}) (d) based on thermally uncoupled levels.
